# The impact of tissue characterization for in-stent restenosis with optical coherence tomography during excimer laser coronary angioplasty

**DOI:** 10.1007/s12928-018-0543-8

**Published:** 2018-08-22

**Authors:** Sho Hashimoto, Akihiko Takahashi, Yukio Mizuguchi, Takeshi Yamada, Norimasa Taniguchi, Tetsuya Hata, Shunsuke Nakajima

**Affiliations:** Department of Cardiology, Sakurakai Takahashi Hospital, 5-18-1 Oikecho, Sumaku, Kobe, Hyogo 654-0026 Japan

**Keywords:** Excimer laser coronary angioplasty, In-stent restenosis, Optical coherence tomography

## Abstract

We aimed to evaluate the impact of tissue characterization for in-stent restenosis (ISR) with optical computed tomography (OCT) during excimer laser coronary angioplasty (ELCA) in the drug-eluting stent (DES) era. The effect of ELCA for ISR according to differences in tissue characteristics is unclear. Fifty-three ISR lesions (7 bare metal stents and 46 drug-eluting stents) were treated with an ELCA catheter. After ELCA, balloon dilatation with either the scoring or non-compliant balloons was conducted. The procedure was completed by applying a drug-coated balloon. Tissue characterization and lumen measurement with OCT were performed thrice: (1) before percutaneous coronary intervention (PCI), (2) after ELCA, and (3) and after the procedure. Lesions were categorized into the homogenous, layered, and mixed groups. Follow-up angiograms were conducted 6–12 months after PCI. No significant differences in minimal lumen area (MLA) were observed before PCI. A significant difference was observed in MLA after ELCA among the three groups (homogeneous group: 1.75 ± 0.84 mm^2^, layered group: 1.72 ± 0.45 mm^2^, mixed group: 2.24 ± 0.70 mm^2^, *P* = 0.048). Final MLA was larger in the mixed group than in the homogeneous group (*P* = 0.028). No significant difference was observed in binary restenosis in the follow-up angiogram (homogeneous group 55.5%, layered group 33.3%, mixed group 33.3%; *P* = 0.311) and the target lesion revascularization rate (homogeneous 30.0%, layered 23.8%, mixed 25.0%; *P* = 0.923). Tissue characterization by OCT may predict the efficacy of ELCA and balloon angioplasty for ISR during the acute phase.

## Introduction

The introduction of the drug-eluting stent (DES) in percutaneous coronary intervention has significantly reduced the incidence of in-stent restenosis (ISR) [1, 2]. Further, new generation DES has shown superior clinical results in patients with acute coronary syndrome (ACS) in terms of stent thrombosis. Therefore, DES is more frequently used for a wide spectrum of patients with coronary artery disease. However, the incidence of repeat revascularization still exceeds 10% in real-world patients with complex lesions [3], even with the widespread use of DES. Therefore, the treatment of patients with ISR remains challenging. Although several treatments have been proposed, no definite solution has been established. Indeed, a few patients require multiple percutaneous coronary interventions (PCI) with additional DES implantation, drug-coated balloon, and/or scoring balloon catheters.

The efficacy of excimer laser coronary angioplasty (ELCA) (Spectranetics, Advanced Interventional Systems) for the treatment of ISR with bare metal stent (BMS) has been evaluated with angiographic or intravascular ultrasound (IVUS) guidance; however, these studies failed to show superiority over conventional balloon angioplasty in the chronic phase [4, 5].

With respect to ISR with DES, recent studies have shown the existence of a variety of materials that can contribute to the completion of ISR. However, there is a lack of sufficient data indicating a difference in the efficacy of ELCA for the treatment of ISR according to tissue characteristics. Optical coherence tomography (OCT) is a new imaging modality that visualizes intracoronary features with an axial resolution of 3–20 μm, which is substantially higher than that of IVUS (100–150 μm) and enables tissue characterization compatible with the findings of histopathological examinations. Accordingly, in this study, we evaluated the impact of tissue characterization for ISR with OCT during ELCA in the DES era.

## Methods

### Study design and population

Between April 2014 and August 2017, records of 39 consecutive patients (53 lesions) who underwent ELCA under OCT guidance for ISR and follow-up angiograms were retrospectively analyzed. After ELCA ablation, an additional balloon dilatation was conducted. The procedure was finalized by the application of a drug-coated balloon. During PCI, tissue characterization and lumen measurement with OCT were performed at 3 time points: (1) before PCI, (2) after ablation with ELCA, and (3) at the end of procedure. Based on initial OCT findings, the lesions were categorized into 3 groups: (1) homogenous, (2) layered, and (3) mixed group. For each lesion, follow-up angiograms were conducted at 6–12 months (10.1 ± 5.0 months) after the PCI.

### PCI procedure

PCI was performed using a 5- or 6-Fr guiding catheter. Intravenous heparin was administered to maintain an activated clotting time of > 300 s. OCT evaluation was conducted at each stage of the procedure, i.e., before and after ELCA, and at the end of the procedure. After ELCA, scoring balloon and/or non-compliant balloon dilatation as well as application of drug-coated balloon were performed in all cases. Adequate balloon size was determined at the operator’s discretion by considering the size of the old stent as well as angiographic and OCT findings.

### ELCA procedure

In the 53 lesions treated with ELCA, the procedure was performed as described elsewhere [4–8]. The selection of laser catheter size was at the operator’s discretion based on the OCT findings. Sixteen lesions were treated using a 0.9-mm laser catheter and 37 lesions were treated using a 1.4-mm catheter.

The first pass of ELCA was performed at a fluence of 45 mJ/mm^2^ and a repetition rate of 25 Hz. The maximum fluence used in the ablation was 60 mJ/mm^2^ and the maximum repetition rate was 40 Hz. The endpoint of ELCA was determined at the operators’ discretion, mainly by (1) formation of a dissection, which may cause a larger dissection, requiring stent implantation by further ablation; (2) exposure of a stent strut in the lesion; and (3) a long ablation time (> 10 min).

### OCT procedure and analysis

OCT was performed in all patients with the Dragonfly™ OPTIS™ Imaging Catheter (St. Jude Medical, St Paul, MN, USA) using a non-occlusive technique and flushed with undiluted contrast or low molecular weight dextran. Motorized OCT pullbacks were performed at a rate of 36 mm/s, and all images were acquired at 180 frames per second.

OCT quantitative analysis was performed according to the agreement of two experienced analysts, blinded to clinical characteristics and classified the group using commercial software (St. Jude Medical) at every 1 mm cross-section throughout the pullback from the distal to the proximal stent edge. The minimal lumen area (MLA) was measured in the beginning, after ELCA, and at the end of the procedure. Stent area (SA) was measured at the same cross-section of MLA in initial and final OCTs. Neointimal area (NA) was calculated as the incremental difference between the SA and MLA. Acute lumen gain was calculated as the incremental difference between MLA measured after ELCA and initial MLA. Final lumen gain was calculated as the incremental difference between the final and initial MLAs. Percent stent dilatation was calculated as Final SA/initial SA × 100. Percent neointimal reduction was calculated as (1 − final NA/initial NA) × 100.

**Fig. 1:**
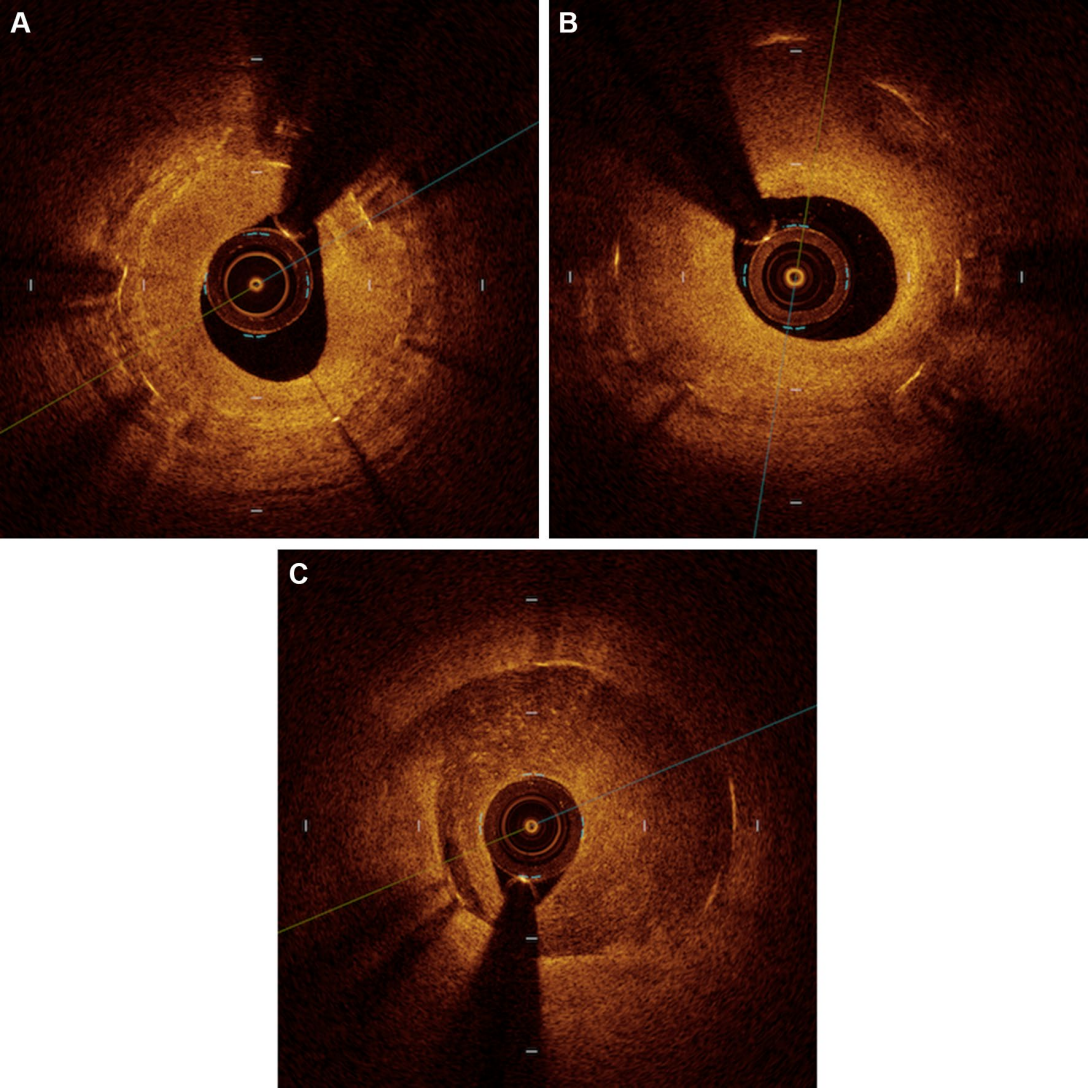
Representative cases showing each type of tissue morphology as assessed by OCT. **a** homogeneous type; **b** layered type; **c** mixed type

Qualitative OCT analysis was also performed according to the agreement of two experienced analysts who were not quantitative analysts. The neointimal tissue, including MLA in the initial OCT, was classified as (1) homogeneous tissue, i.e., a uniform signal-rich band without focal validation or attenuation; (2) layered tissue, i.e., concentric layers with different optical properties; and (3) mixed tissue, i.e., focally changing optical properties and various backscattering patterns [6]. Representative cases with each type of tissue are shown in Fig. 1.

### Statistical analysis

Categorical data were compared using *χ*^2^ statistics or Fisher’s exact test. Data with continuous variables were expressed as numbers (%) or mean ± standard deviation. Continuous variables between 2 groups were compared using Student’s *t* test or the Mann–Whitney *U* test. Continuous variables among the three groups were compared using analysis of variance (ANOVA) or the Kruskal–Wallis test and the Tukey test or Steel–Dawass test was used as post hoc analysis when a significant difference was found using ANOVA or the Kruskal–Wallis test. Additionally, to eliminate the influence of potential covariates, the analysis of covariance (ANCOVA) test was used to analyze continuous variables among the three groups when there were differences of variables among the baseline groups. Multiple lesions within the same patient were assumed to be independent of each other. *P* < 0.05 was considered statistically significant. All statistical analyses were performed using EZR (Easy R, Saitama Medical Center, Jichi Medical University; Kanda, 2013), which is a graphical interface for R (The R Foundation for Statistical Computing, Vienna, Austria, Version 3.0.2).

## Results

### Baseline patient and lesion characteristics

The patients included 24 men and 15 women with a mean age of 72.6 ± 8.7 years. Of the total 53 lesions, 20 were in the homogeneous group, 21 in the layered, and 12 in the mixed group. Baseline patient characteristics and lesion characteristics of the three groups are summarized in Tables 1 and 2. There was no significant difference in the type of stent and terms after stenting among the three groups. In the PCI procedure, the maximum size of balloon catheter and the size of drug-coated balloon (DCB) in the homogeneous group were significantly smaller than those in the other groups. The size of ELCA catheter, maximum energy densities, and total pulse number were similar among the groups. All procedures were successfully conducted without major complications including death, myocardial infarction, and coronary perforation.

**Table 1:** Baseline patient characteristics

	*N* = 39
Age	72.6 ± 8.7
Male gender (%)	24 (61.5)
Body height (cm)	160.5 ± 8.5
Body weight (kg)	60.8 ± 10.2
Coronary risk factors	
Hypertension (%)	31 (79.8)
Dyslipidemia (%)	36 (92.3)
Diabetes mellitus (%)	16 (41.0)
Current smoking (%)	5 (12.8)
Medications	
Statin (%)	35 (89.7)
ACE-I/ARB (%)	23 (58.9)
β-blocker (%)	22 (56.4)
CCB (%)	20 (51.2)
Anticoagulant (%)	1 (2.5)
DAPT (%)	39 (100)

*ACE-I* angiotensin converting enzyme inhibitor, *ARB* angiotensin II receptor blocker, *CCB* calcium channel blocker, *DAPT* dual antiplatelet therapy

**Table 2:** Baseline lesion characteristics and PCI procedure

	Homogeneous *n* = 20	Layered *n* = 21	Mixed *n* = 12	*P* value
Culprit lesion				
RCA (%)	6 (30.0)	13 (61.9)	9 (75.0)	0.027
LAD (%)	12 (60.0)	7 (33.3)	3 (25.0)	0.093
LCX (%)	2 (10.0)	1 (4.7)	0 (0.0)	0.482
				0.08*
Stent type				
BMS (%)	3 (15.0)	2 (9.5)	2 (16.6)	0.774*
DES (%)	17 (85.0)	19 (90.5)	10 (83.4)	
First generation DES (%)	8 (40.0)	6 (28.5)	2 (16.6)	0.371
Second generation DES (%)	9 (45.0)	13 (61.9)	8 (66.6)	0.4
Duration after stenting (months)	45.4 ± 37.5	45.1 ± 35.0	51.9 ± 53.1	0.883
Stent size (mm)	2.66 ± 0.35	3.00 ± 0.44	3.04 ± 0.43	0.014
Stent length (mm)	24.2 ± 7.7	23.4 ± 8.1	18.8 ± 5.3	0.125
Procedure				
Guiding catheters				
Use of 5Fr Guiding Catheter (%)	2 (10.0)	1 (4.7)	2 (16.6)	0.525
ELCA catheters				
0.9 mm ELCA catheter (%)	8 (40.0)	5 (23.8)	3 (25.0)	0.514*
1.4 mm ELCA catheter (%)	12 (60.0)	16 (66.2)	9 (75.0)	
Maximum energy densities (mJ/mm2)	56.3 ± 8.7	59.1 ± 3.0	57.5 ± 5.8	0.37
ELCA pulse number	4234 ± 2074	4204 ± 1714	4029 ± 1398	0.948
Maximum balloon size (mm)	2.76 ± 0.52	3.02 ± 0.45	3.23 ± 0.39	0.03
Balloon length (mm)	12.9 ± 1.4	12.9 ± 0.8	12.3 ± 1.3	0.29
Maximum balloon pressure (atm)	15.7 ± 4.7	15.8 ± 4.7	17.2 ± 4.8	0.66
DCB size (mm)	2.86 ± 0.46	3.13 ± 0.48	3.30 ± 0.39	0.019
DCB length (mm)	21.9 ± 5.8	19.5 ± 5.8	18.8 ± 5.6	0.55
DCB inflation time (s)	54.0 ± 10.6	46.0 ± 14.5	55.4 ± 8.9	0.05
Duration to f/u CAG (months)	10.1 ± 4.9	10.1 ± 5.3	9.9 ± 4.9	0.989

*RCA* right coronary artery, *LAD* left anterior descending artery, *LCX* left circumflex artery, *DES* drug-eluting stent, *ELCA* excimer laser coronary angioplasty, *DCB* drug-coated balloon

*P values from Fisher’s exact test for 3 × 2 or 3 × 3 contingency table

### OCT analysis

OCT findings in each stage during PCI are listed in Table 3. No significant difference was observed in the initial MLA (1.01 ± 0.31 mm^2^, 1.06 ± 0.50 mm^2^, 1.29 ± 0.75 mm^2^, respectively; *P* = 0.821). Initial stent area in the homogeneous group (5.06 ± 1.65 mm^2^) was significantly smaller than that in the layered group (6.85 ± 2.17 mm^2^, *p* = 0.033) and in the mixed group (6.82 ± 1.51 mm^2^, *P* = 0.033). A significant difference was observed in MLA after ELCA among three groups (homogeneous group: 1.75 ± 0.84 mm^2^, layered group: 1.72 ± 0.45 mm^2^, mixed group: 2.24 ± 0.70 mm^2^, *P* = 0.048). Final MLA in the homogeneous group (4.03 ± 1.46 mm^2^) was smaller than that in the layered group (4.92 ± 1.79 mm^2^, *P* = 0.195) and in the mixed group (5.49 ± 1.45 mm^2^, *P* = 0.028). Similarly, final stent area in the homogeneous group (6.68 ± 2.42 mm^2^) was smaller than that in the layered (8.54 ± 2.53 mm^2^, *P* = 0.05) and mixed groups (8.85 ± 2.21 mm^2^, *P* = 0.045). There was no significant difference in percent stent dilatation (homogeneous: 133.9%, layered: 126.1%, mixed: 131.5%, *P* = 0.652) and percent neointimal reduction (homogeneous: 35.3%, layered: 38.4%, mixed: 38.3%, *P* = 0.932) among the three groups. As a significant difference was observed in the initial stent area among the three groups, ANCOVA analysis with the initial stent area as a covariate was performed, showing no significant differences among the groups (Table 4).

**Table 3:** OCT findings and prognosis

	Homogeneous *n* = 20	Layered *n* = 21	Mixed *n* = 12	*P* value	H vs L	H vs M	L vs M
Initial MLA (mm^2^)	1.01 ± 0.31	1.06 ± 0.50	1.29 ± 0.75	0.821	–	–	–
After ELCA MLA (mm^2^)	1.75 ± 0.84	1.72 ± 0.45	2.24 ± 0.70	0.048	0.745	0.063	0.106
Final MLA (mm^2^)	4.03 ± 1.46	4.92 ± 1.79	5.49 ± 1.45	0.036	0.195	0.028	0.712
Initial stent area (mm^2^)	5.06 ± 1.65	6.85 ± 2.17	6.82 ± 1.51	0.013	0.033	0.033	0.892
Final stent area (mm^2^)	6.68 ± 2.42	8.54 ± 2.53	8.85 ± 2.21	0.021	0.05	0.045	0.996
Acute lumen gain (mm^2^)	0.73 ± 0.91	0.65 ± 0.41	0.94 ± 0.39	0.025	0.91	0.019	0.089
Final lumen gain (mm^2^)	3.01 ± 1.63	3.86 ± 1.59	4.20 ± 1.68	0.073	–	–	–
Stent dilatation (mm^2^)	1.62 ± 1.25	1.68 ± 1.21	2.02 ± 1.54	0.811	–	–	–
% Stent dilatation	133.9 ± 24,4	126.1 ± 20.6	131.5 ± 24.0	0.652	–	–	–
% Neointimal reduction	35.3 ± 14.3	38.4 ± 16.1	38.3 ± 21.9	0.932	–	–	–
ISR (%)	11 (55.5)	7 (33.3)	4 (33.3)	0.311*	0.64	0.87	1
No ISR (%)	9 (44.5)	14 (66.7)	8 (66.7)		0.64	0.87	1
TLR (%)	6 (30.0)	5 (23.8)	3 (25.0)	0.923*	1	1	1
No TLR (%)	14 (70.0)	16 (76.2)	9 (75.0)		1	1	1

*MLA* minimal lumen area, *ELCA* excimer laser coronary atherectomy, *ISR* in-stent restenosis, *TLR* target lesion revascularization, *H* homogeneous, *L* layered, *M* mixed

*P values from Fisher’s exact test for 3 × 2 contingency table

**Table 4:** ANCOVA analysis (ANCOVA analysis performed with covariate of initial stent area)

Dependent variables	*Df*	SS	*F* value	*P* value
After ELCA MLA (mm^2^)	2	1.979	2	0.104
Final MLA (mm^2^)	2	3.128	0.829	0.443
Final stent area (mm^2^)	2	0.844	0.247	0.782
Acute lumen gain (mm^2^)*	–	–	–	–
Final lumen gain (mm^2^)	2	1.398	0.330	0.72
Stent dilatation (mm^2^)	2	0.844	0.247	0.782
% Stent dilatation	2	0.166	3.330	0.819
% Neointimal reduction	2	0.024	0.418	0.661

*MLA* minimal lumen area, *ELCA* excimer laser coronary atherectomy, *Df* degree of freedom, *SS* sum of square

*Significant interaction was observed between acute lumen gain and a covariate (initial stent area)

### Clinical outcomes at the chronic phase

The follow-up angiogram revealed no significant differences in terms of binary angiographic restenosis (homogeneous 55.5%, layered 33.3%, mixed 33.3%; *P* = 0.311) and the target lesion revascularization rate (TLR; homogeneous 30.0%, layered 23.8%, mixed 25.0%; *P* = 0.923).

## Discussion

The current study demonstrated that OCT examination for in-stent restenosis presents heterogeneity in the components including homogenous (*n* = 20), layered (*n* = 21), and mixed groups (*n* = 12), which is similar to observations from recent studies [9, 10]. There were significant differences in MLA and lumen gain among the three groups after ELCA. Furthermore, a significantly larger MLA was observed in the mixed group than in the homogenous group in final OCT. Although a significant difference was observed in the initial stent area among the three groups, ANCOVA analysis with the initial stent area as a covariate did not show any significant difference among the groups in terms of outcomes including MLA after ELCA, final MLA, final stent area, acute lumen gain after ELCA, final lumen gain, stent dilatation, and percent stent dilatation. Despite the difference in final MLA, the incidences of binary in-stent restenosis and TLR showed no statistically significant difference among the groups.

Although ISR is less common after introduction of the DES, the treatment of ISR still remains a challenge [11]. It usually presents as recurrent angina and has often been managed by repeat percutaneous revascularization including balloon angioplasty with either a non-compliant or scoring balloon, DCB angioplasty, additional DES implantation, and surgical revascularization. Although such options have been recommended as a treatment solution of ISR, none of them are considered to be definitive.

ELCA is designed to ablate the obstructive atherosclerotic plaque rather than creating deformation of the plaque as in balloon dilatation. This design is considered as a more rational treatment option for debulking before adjunctive balloon dilatation, in contrast to balloon dilatation alone for stent restenosis, as it is superior in dilating the lumen and stent areas [12]. In fact, ELCA with an IVUS-based study for ISR in bare metal stents was documented to be effective in the ablation of neointimal tissue in BMS [13]. However, acute procedural results as well as long-term angiographic and clinical results of ELCA with balloon dilatation were not superior to balloon dilatation alone [12, 13]. Accordingly, ELCA was abandoned as a strategy for treating ISR. Moreover, according to the recent guidelines on myocardial revascularization, i.e., European Society of Cardiology Guideline, ELCA was not cited as useful for the treatment of in-stent restenosis.

Recent research on pathological examination of human specimens indicated that accumulated tissue contributing to ISR after stent implantation consists of various components, including proteoglycan-rich tissue, thrombi, smooth muscle cells, atheromas, inflammatory cells, and fibrinoids. These differences in components can reflect those in OCT findings; a homogeneous pattern is likely composed of smooth muscle cells with collagen, whereas the main components of layered and mixed patterns likely include proteoglycans, cell matrix, fibrinoids, and organized thrombi [9]. Furthermore, a recent study indicated that the layered pattern represents ISR comprising proteoglycan-rich tissue with smooth muscle cells richly distributed on the luminal side, and the mixed pattern indicates neointimal tissues comprising of organized thrombus and/or fibrinoids with smooth muscle cells which are poorly and focally distributed [9].

Considering these differences in the components of ISR tissues, the responses of tissue to treatment with balloon angioplasty were reported to be different. In the lesions with homogenous findings on OCT, a change in the luminal cross-sectional area was smaller and the stent cross-sectional area was larger than those of the non-homogenous group. Thus, ISR lesions with homogenous neointima are associated with a relative overexpansion of the stent with suboptimal compression of the neointima during intervention.

In the current study, the mixed group showed a significantly higher efficacy of ELCA and subsequent balloon angioplasty compared to the homogenous pattern group. These findings are compatible with the results of balloon angioplasty in recent studies [7, 9, 10, 14]. Furthermore, lumen gain in the stent area was almost the same among the groups. Compared to simple balloon dilatation, ELCA may contribute to prevent stent overexpansion as there was no difference in percentage stent expansion among the three groups.

There is paucity of data elucidating a correlation between OCT findings and chronic results of ISR treatment with DCB and/or ELCA. In a recent report using DCB monotherapy (without ELCA) for ISR, the incidence of angiographic re-restenosis was 19.1% in homogeneous, 22.0% in layered, and 38.5% in mixed groups, and the TLR rate was 10.6%, 18.3%, and 34.6%, respectively [10]. In the current study, the incidence of angiographic re-restenosis was 56% in homogeneous, 33% in layered, 33% in mixed group, and TLR rate was 30.0%, 23.8%, and 25.0%, respectively. Most major differences in the incidence of ISR and TLR between the two studies were observed between the homogenous type groups. These differences may partly reflect the fact that original stent and balloon size in the current study were significantly smaller in the homogeneous group than in the remaining groups. Nonetheless, the initial MLA before ELCA was similar among three groups; therefore, ELCA ablation before DCB may cause adverse tissue response to treatment in the homogenous group. Conversely, the benefit of ELCA in the layered and mixed group was equivocal. A possible explanation for such conflicting results in the chronic phase according to the tissue characteristics likely includes the differences in the inflammatory changes in the smooth muscle cells due to heat injury and subsequent healing process in the remaining tissue after ELCA. Further, an occasional strut exposure to the vessel lumen because of excessive ablation may also play a role in the development of ISR regardless of tissue morphology. Thus, although the clinical results of chronic phase in the current study did not show any favorable results, tissue characterization using OCT may provide information for differentiating the effect of ELCA and DCB for ISR. Further studies on larger sample sizes are warranted in terms of patient selection for ELCA therapy.

This study has several limitations. First, this was a single-center, retrospective, observational study. Only 53 lesions with 3 cohorts were evaluated. Thus, the study’s ability to detect significant correlates of effects with ELCA and OCT finding was limited. Second, there was no control group to evaluate the additional effect of ELCA in ISR treatment. Third, other potential confounders that could affect the result, such as the difference of the diseased stent (BMS or DES, the generation of DES, stent size, stent length), balloon size, and differences in the position of the coronary stent, were not evaluated. In fact, the homogenous group utilized a significantly smaller balloon size and stent used in the index procedure. Fourth, the risk factors contributing to ISR and TLR, such as the presence of antiplatelet therapy, ACS presentation, older age, diabetes mellitus, and multiple TLR, were not fully evaluated.

## Conclusion

For the treatment of ISR, the efficacy of ELCA during the acute phase depends on tissue characteristics as detected by OCT. Despite these differences observed in the acute phase, no clinical difference in the chronic phase was observed. Further studies with a larger sample size are warranted.
